# Supplementary material for: Future trends in incidence and long-term survival of metastatic cancer in the United States
Source: Commun Med (Lond). 2023 May 27;3:76. doi: 10.1038/s43856-023-00304-x (PMC10224927; doi:10.1038/s43856-023-00304-x)
Supplement: Supplementary file 1 — Description of Additional Supplementary Files [file 43856_2023_304_MOESM1_ESM.pdf]

Description of Additional Supplementary File

Supplemental Data 1. SEER Output

SEER Output provides the raw output from SEER Stat. Information to replicate this exact output is provided in the “info” tab. Information to access SEER is provided in Supplemental Information.

Supplemental Data 2. Arima Model Output

Arima Model Output provides output from APC ARIMA forecast. Input was derived from SEER Output. All data and figures were derived from APC ARIMA forecast output. More information on this model is available in Supplemental Information.

Supplemental Data 3. SEER Mets to Brain, Bone, Liver, Lungs

SEER Mets to Brain, Bone, Liver, Lungs provides the raw output from SEER Stat. Information to replicate this exact output is provided in the “info” tab corresponding to the individual cancer. Instructions to install SEER is provided in Supplemental Information.

Supplemental Data 4. Joinpoint Template

Joinpoint Template provides the template for the inputs to our Joinpoint calculations. After opening incidence file on Joinpoint, under “Input File” tab, “Dependent Variable,” select “Type of Variable,” “Crude Rate.” Individually select cancers by incidence under “Crude Rate” drop box. Under “Independent Variable,” select year column, and “Interval Type,” “Annual.” Under “Heteroscedastic/Correlated Errors Option,” select “Constant Variance,” then mark “First Order Autocorrelation estimated from the data.” Under “Method and Parameters” tab, “Number of Joinpoints” change “maximum” to 3. All other settings are unchanged from stock.

Supplemental Data 5. Mets to Bone

Mets to Bone provides the APC ARIMA forecast for SEER metastasis to bone. These are incidence of metastatic lesions from the given primary site to bone.

Supplemental Data 6. Mets to Brain

Mets to Brain provides the APC ARIMA forecast for SEER metastasis to bone. These are the incidence of metastatic lesions from the given primary site to the brain.

Supplemental Data 7. Mets to Liver

Mets to Liver provides the APC ARIMA forecast for SEER metastasis to liver. These are the incidence of metastatic lesions from the given primary site to the liver.

Supplemental Data 8. Mets to Lung

Mets to Lung provides the APC ARIMA forecast for SEER metastasis to lung. These are the incidence of metastatic lesions from the given primary site to the lungs.

Supplemental Data 9. Case Listing File

The Case Listing File is the raw dataset that provides the publicly available SEER output that was used to calculate the odds ratios in Figure 4. This was also used for the Jump model.
