# Supplementary material for: Future trends in incidence and long-term survival of metastatic cancer in the United States
Source: Commun Med (Lond). 2023 May 27;3:76. doi: 10.1038/s43856-023-00304-x (PMC10224927; doi:10.1038/s43856-023-00304-x)
Supplement: Supplementary file 11 — Supplementary Information [file 43856_2023_304_MOESM11_ESM.docx]

**Supplemental Online Content**

Future trends in incidence and long-term survival of metastatic cancer in the United States

**Supplemental Methods.** Surveillance, Epidemiology, and End Results (SEER) Database

**Supplemental Figure 1.** Joinpoint analysis of all metastatic cancer subtypes, 1988-2040

**Supplemental Figure 2.** Joinpoint analysis of metastatic cancer to common cancer sites, 2010-2040

**Supplemental Figure 3.** Forecasting age demographic incidence of metastatic cancer, 1988-2040

**Supplemental Table 1.** Incidence and count of metastatic cancer patients, 1988-2040

**Supplemental Table 2.** Changes in long-term survivorship across all metastatic cancer subtypes, 1988-2040

**Supplemental References.**

This supplementary material has been provided by the authors to give readers additional information about their work.

**Supplemental Methods.** Surveillance, Epidemiology, and End Results (SEER) Database

***SEER registry selection***

Detailed description of SEER registries are found below. For the purposes of this study, authors elected to use SEER 9 registry for data on metastatic patients, and long-term survivors of metastatic disease, because this registry offered the largest range of years, 1975-2018. Newer registries included greater number of area’s but lacked the time series data on population and metastatic patients necessary to make the most accurate prediction models available. SEER 18 registry was used for data of metastatic patients with metastases to the brain, bone, liver, or lungs. SEER 18 was used as these variables were only available from 2010+, and thus captured more patients using a database of greater area. To examine metastatic disease to a location; metastases to the brain, bone, liver, or lungs variables were the only available at the time of this study.

***Calendar period, registry, and diversity***

The SEER program has evolved since its inception in the United States in 1973^1^. As of 2017, SEER has up to 36 years of longitudinal and ongoing data collection, with a representative sample size of more than 6 million cancer cases, and a comprehensive quality assurance process. Over time, more registries were added to SEER; in the current analysis, the SEER 9 and 18 (adjusted for Hurricane Katrina Impacted Louisiana cases) were used. The registry number denotes the number of registries. *SEER 9.*The first areas included at that time were Connecticut, Hawaii, Iowa, San Francisco/Oakland, and Detroit. Geographic areas were included based on two objectives: (1) the ability of a geographic cancer registry to maintain high-quality data (explained below), and (2) having a population that represents minority subpopulations^1^.

Since the SEER database have increased the proportion of the US population captured over the years, in early years of the SEER program there are fewer survivors than in later years, and the proportion of death by index cancer is lower in later years. Further, the rate count of people having a cancer depends on the number of patients living with this cancer from previous years (which depends on cancer prevalence), those diagnosed within the calendar year (which depends on screening and incidence), and those dying during that year (which depends on cancer and treatment aggressiveness, how death is coded, common risk factors among cancers and comorbidities, and patient age). Certain cancers have an indolent course (e.g. prostate), and patients diagnosed in subsequent years are added to the cumulative count, increasing the number of prostate cancer patients relative to all others; for patients with aggressive cancers (e.g. pancreatic), the addition of patients diagnosed in subsequent years has little effect on the cumulative number because of high rates of mortality.

***Quality assurance and completeness***

SEER undergoes quality assurance using systematic, standardized, and periodic data collection procedure for all defined members of a defined cohort is performed to avoid surveillance bias.^1^ The case-finding audits are performed by a qualified member from each SEER registry under the direction of members of the National Cancer Institute. Auditors create an abstract the contains the primary site and the case finding source^2^. When performing audits, SEER adheres to two basic principles: auditing high quantity and high-risk data. High quantity refers to disease sites that have the highest incidence and prevalence (e.g. breast, prostate, lung, colon); as well facilities that contribute the greatest percent of cases to the central database. Additionally, pathology laboratories are selected to review tissue from patients not seen at that hospital. High risk refers to cases that are likely to be miscoded (e.g. head and neck, hematopoietic diseases); compliance to new rules; and newly-reportable diseases.

***Data availability statement (DAS)***

The instructions to access the SEER data are provided below:

(1) Download the SEER*Stat software from the NCI website: [https://seer.cancer.gov/seerstat/software/](about:blank)

(2) Open the program

(3) Click “File”, “New,”

**“Incidence Session”** to generate a list of the incidence rates of cancers.

(4) Click on the desired registry to use for each of the sessions. Specific inputs to replicate this data selection can be found in **Supplemental Data 1** “SEER Output,” and **Supplemental Data 3** “SEER Mets to Brain, Bone, Liver, Lungs,” under the individual “info” tabs. **Supplemental Data 2** “ARIMA Model Output” contains data extrapolated to create Figures 1 and 3, Supplemental Figures 1 and 3, Table 1 (top), and Supplemental Table 1. **Supplemental Data 5-8** “Mets to ….” contain data extrapolated to create Figure 2, Supplemental Figure 2, and Table 1 (bottom). **Supplemental Data 9** contain the SEER case listing file used to produce Figure 4. All data can also be accessed and retrieved publicly from the NCI website, as outlined in steps 1-3 above.

***Code availability statement (CAS)***

The Joinpoint template used in this paper can be accessed in **Supplemental Data 3.**

**
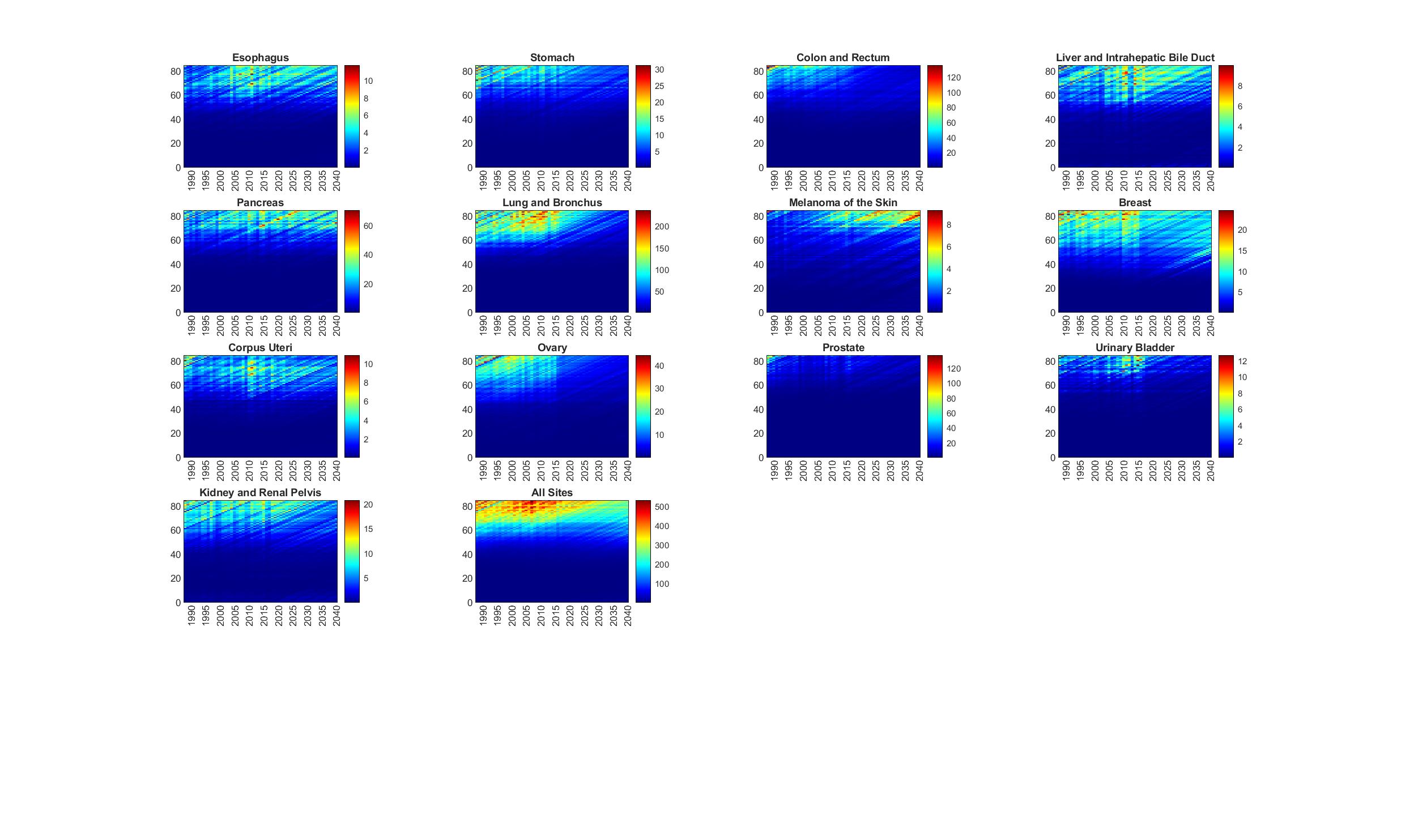
**

**Supplemental Figure 1. Forecasting age demographic incidence of metastatic cancer, 1988-2040.**

Heat map of Historical and predicted metastasis incidence per 100,000 for individuals ages 0-84 years (y axis) for the years 1988-2040 (x axis). Each plot indicates a different origin tumor of metastasis. Red implies high incidence while blue is lower. Colors are represented numerically by the scale bar to the right of each graphic.


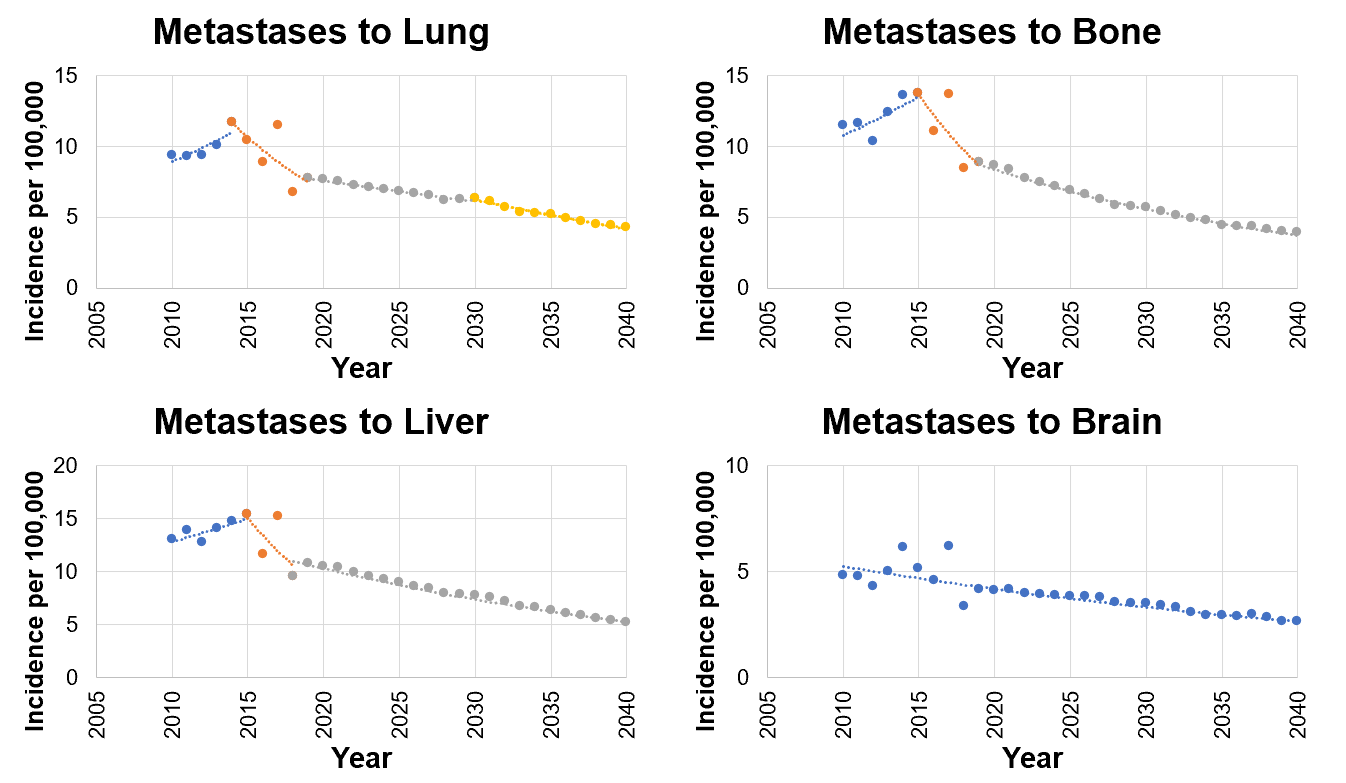


**Supplemental Figure 2. Join point analysis of metastatic cancer to common cancer sites, 2010-2040**

Join Point analysis (up to three JoinPoints allowed) of trends of ARIMA predicted metastatic cancer incidence to a specific site. Individual line segments represent unique significant trends in metastatic cancer. Shown is the predicted trend of all primaries to that site, but does not describe changes in incidence of individual primary cancers metastasis to each site.


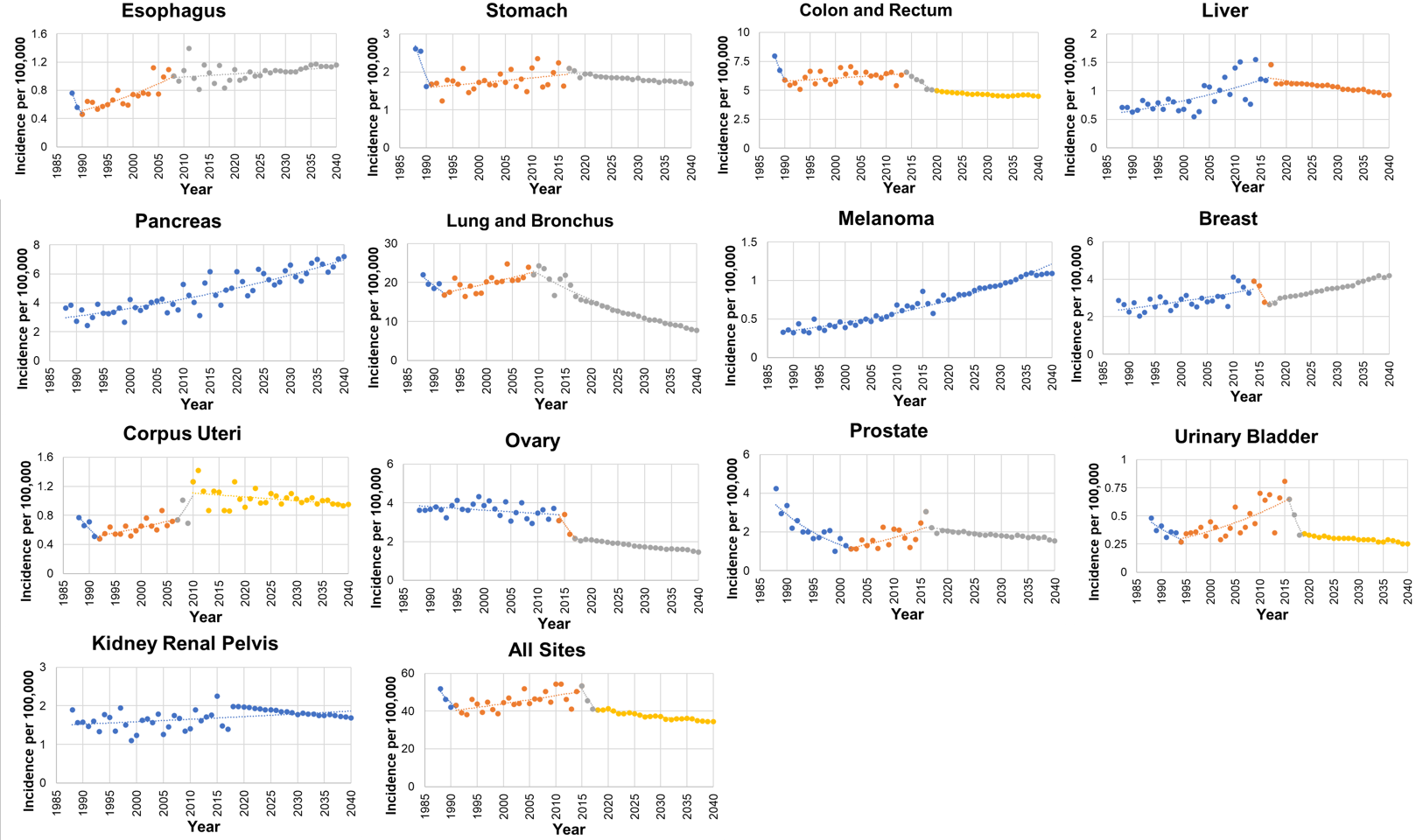


**Supplemental Figure 3. Joinpoint analysis of all metastatic cancer subtypes, 1988-2040**

Join Point analysis (up to three JoinPoints allowed) of trends of ARIMA predicted age-standard metastatic cancer incidence rates, by tumor origin. Individual line segments represent unique significant trends in metastatic cancer. The predicted trends of all metastatic cancer do not reflect the trends of individual diseases.

| **ARIMA** | 1990 | | 1995 | | 2000 | | 2005 | | 2010 | | 2015 | |
| --- | --- | --- | --- | --- | --- | --- | --- | --- | --- | --- | --- | --- |
| Primary cancer site | *N* | *Rate* | *N* | *Rate* | *N* | *Rate* | *N* | *Rate* | *N* | *Rate* | *N* | *Rate* |
| All | 9,856 | 42.160 | 10,950 | 43.791 | 11,746 | 44.499 | 11,956 | 43.927 | 15,465 | 54.289 | 15,842 | 53.330 |
| Lung and Bronchus | 4,334 | 18.539 | 4,855 | 19.418 | 5,324 | 20.171 | 5,576 | 20.486 | 6,901 | 24.228 | 6,504 | 21.896 |
| Colon and Rectum | 1,374 | 5.878 | 1,660 | 6.640 | 1,522 | 5.764 | 1,538 | 5.650 | 1,830 | 6.426 | 1,843 | 6.203 |
| Prostate | 787 | 3.368 | 415 | 1.661 | 439 | 1.662 | 351 | 1.289 | 609 | 2.138 | 733 | 2.467 |
| Pancreas | 636 | 2.721 | 822 | 3.288 | 1,114 | 4.221 | 1,128 | 4.143 | 1,503 | 5.276 | 1,830 | 6.161 |
| Ovary | 859 | 3.672 | 1,033 | 4.132 | 1,021 | 3.868 | 832 | 3.056 | 987 | 3.466 | 1,009 | 3.397 |
| Breast | 528 | 2.257 | 632 | 2.529 | 774 | 2.933 | 762 | 2.799 | 1,173 | 4.116 | 1,087 | 3.661 |
| Stomach | 378 | 1.616 | 440 | 1.760 | 454 | 1.720 | 468 | 1.718 | 601 | 2.112 | 667 | 2.244 |
| Kidney and Renal | 370 | 1.583 | 426 | 1.704 | 325 | 1.233 | 343 | 1.260 | 403 | 1.414 | 668 | 2.250 |
| Corpus Uteri | 166 | 0.710 | 135 | 0.539 | 173 | 0.655 | 180 | 0.660 | 359 | 1.260 | 332 | 1.118 |
| Esophagus | 108 | 0.460 | 150 | 0.601 | 196 | 0.744 | 203 | 0.746 | 307 | 1.078 | 313 | 1.053 |
| Liver | 146 | 0.626 | 198 | 0.792 | 180 | 0.682 | 292 | 1.072 | 398 | 1.398 | 360 | 1.212 |
| Urinary Bladder | 97 | 0.413 | 86 | 0.344 | 120 | 0.454 | 157 | 0.575 | 200 | 0.702 | 240 | 0.808 |
| Melanoma | 74 | 0.317 | 95 | 0.382 | 103 | 0.391 | 129 | 0.472 | 193 | 0.677 | 255 | 0.858 |

| **ARIMA** | 2020 | | 2025 | | 2030 | | 2035 | | 2040 | |
| --- | --- | --- | --- | --- | --- | --- | --- | --- | --- | --- |
| Primary cancer site | *N* | *Rate* | *N* | *Rate* | *N* | *Rate* | *N* | *Rate* | *N* | *Rate* |
| All | 12,737 | 41.316 | 12,355 | 38.649 | 12,314 | 37.194 | 12,370 | 36.108 | 12,285 | 34.448 |
| Lung and Bronchus | 4,597 | 14.911 | 4,044 | 12.651 | 3,602 | 10.881 | 3,203 | 9.348 | 2,782 | 7.801 |
| Colon and Rectum | 1,530 | 4.964 | 1,521 | 4.758 | 1,537 | 4.644 | 1,547 | 4.516 | 1,595 | 4.473 |
| Prostate | 634 | 2.055 | 606 | 1.896 | 596 | 1.799 | 582 | 1.698 | 554 | 1.554 |
| Pancreas | 1,894 | 6.145 | 1,920 | 6.006 | 2,190 | 6.614 | 2,395 | 6.990 | 2,567 | 7.199 |
| Ovary | 641 | 2.079 | 613 | 1.916 | 572 | 1.727 | 556 | 1.623 | 520 | 1.459 |
| Breast | 934 | 3.030 | 1,053 | 3.294 | 1,173 | 3.542 | 1,331 | 3.885 | 1,499 | 4.204 |
| Stomach | 598 | 1.939 | 591 | 1.849 | 606 | 1.831 | 601 | 1.755 | 604 | 1.694 |
| Kidney and Renal | 608 | 1.974 | 604 | 1.890 | 587 | 1.774 | 599 | 1.749 | 603 | 1.690 |
| Corpus Uteri | 281 | 0.912 | 352 | 1.100 | 339 | 1.025 | 342 | 0.999 | 340 | 0.954 |
| Esophagus | 336 | 1.089 | 323 | 1.012 | 351 | 1.060 | 396 | 1.156 | 413 | 1.159 |
| Liver | 351 | 1.140 | 354 | 1.106 | 353 | 1.065 | 353 | 1.032 | 331 | 0.927 |
| Urinary Bladder | 102 | 0.332 | 95 | 0.296 | 96 | 0.289 | 93 | 0.271 | 88 | 0.246 |
| Melanoma | 230 | 0.746 | 279 | 0.874 | 312 | 0.943 | 371 | 1.084 | 388 | 1.089 |

| **SEER** | 1990 | | 1995 | | 2000 | | 2005 | | 2010 | | 2015 | |
| --- | --- | --- | --- | --- | --- | --- | --- | --- | --- | --- | --- | --- |
| Primary cancer site | *N* | *Rate* | *N* | *Rate* | *N* | *Rate* | *N* | *Rate* | *N* | *Rate* | *N* | *Rate* |
| All | 15,669 | 67.021 | 15,387 | 61.536 | 16,622 | 62.971 | 18,626 | 68.431 | 19,748 | 69.327 | 20,798 | 70.016 |
| Lung and Bronchus | 6,432 | 27.514 | 6,830 | 27.314 | 7,401 | 28.038 | 8,636 | 31.727 | 8,537 | 29.971 | 8,269 | 27.836 |
| Colon and Rectum | 2,280 | 9.752 | 2,155 | 8.619 | 2,159 | 8.181 | 2,331 | 8.564 | 2,366 | 8.307 | 2,518 | 8.476 |
| Prostate | 1,584 | 6.777 | 959 | 3.836 | 783 | 2.966 | 777 | 2.856 | 857 | 3.010 | 1,240 | 4.173 |
| Pancreas | 1,073 | 4.590 | 1,140 | 4.558 | 1,367 | 5.178 | 1,579 | 5.800 | 1,772 | 6.222 | 2,033 | 6.845 |
| Ovary | 1,185 | 5.071 | 1,190 | 4.761 | 1,338 | 5.070 | 1,215 | 4.466 | 1,239 | 4.350 | 1,259 | 4.237 |
| Breast | 892 | 3.814 | 907 | 3.629 | 984 | 3.729 | 1,156 | 4.245 | 1,454 | 5.103 | 1,534 | 5.165 |
| Stomach | 659 | 2.817 | 591 | 2.362 | 628 | 2.380 | 695 | 2.554 | 751 | 2.636 | 818 | 2.755 |
| Kidney and Renal | 495 | 2.115 | 541 | 2.162 | 583 | 2.209 | 597 | 2.195 | 646 | 2.267 | 730 | 2.457 |
| Corpus Uteri | 288 | 1.234 | 239 | 0.955 | 283 | 1.072 | 347 | 1.275 | 501 | 1.760 | 565 | 1.901 |
| Esophagus | 274 | 1.172 | 258 | 1.033 | 373 | 1.412 | 451 | 1.656 | 539 | 1.891 | 630 | 2.122 |
| Liver | 212 | 0.906 | 258 | 1.034 | 340 | 1.288 | 371 | 1.362 | 489 | 1.715 | 561 | 1.890 |
| Urinary Bladder | 166 | 0.708 | 154 | 0.616 | 209 | 0.791 | 228 | 0.837 | 298 | 1.047 | 302 | 1.015 |
| Melanoma | 129 | 0.553 | 164 | 0.657 | 173 | 0.656 | 243 | 0.892 | 299 | 1.050 | 340 | 1.144 |

**Supplemental Table 1. Incidence and count of metastatic cancer patients, 1988-2040**

Incidence per 100,000 (Rate), and patient count (N) of metastatic cancers studied. “ARIMA” tables contain values fitted from predictions, whereas “SEER” table contains raw output from the SEER database. Changes in a metastatic cancers’ incidence does not always correlate to proportional increase or decrease in number of patients due to increasing population. The count reflects only the patients of SEER population, not the entirety of the United States.

| **SEER** | 1988 | 1989 | 1990 | 1991 | 1992 | 1993 | 1994 | 1995 | 1996 | 1997 | 1998 | 1999 | 2000 |  |
| --- | --- | --- | --- | --- | --- | --- | --- | --- | --- | --- | --- | --- | --- | --- |
| Primary cancer site | OR | OR | OR | OR | OR | OR | OR | OR | OR | OR | OR | OR | OR |  |
| All | 1 | 0.981 | 1.084 | 1.078 | 1.081 | 1.117 | 1.115 | 1.163 | 1.155 | 1.155 | 1.186 | 1.152 | 1.252 |  |
| Esophagus | 1 | 4.220 | 4.154 | 1.983 | 5.426 | 6.000 | 4.084 | 2.104 | 6.942 | 0.873 | 4.087 | 6.540 | 7.922 |  |
|  |  |  |  |  |  |  |  |  |  |  |  |  |  |  |
| Lung and Bronchus | 1 | 0.967 | 1.056 | 0.977 | 1.174 | 1.169 | 1.351 | 1.136 | 1.230 | 1.215 | 1.459 | 1.327 | 1.395 |  |
|  |  |  |  |  |  |  |  |  |  |  |  |  |  |  |
| Colon and Rectum | 1 | 1.041 | 1.304 | 1.226 | 1.006 | 1.298 | 1.105 | 1.192 | 1.292 | 1.217 | 1.366 | 1.141 | 1.389 |  |
|  |  |  |  |  |  |  |  |  |  |  |  |  |  |  |
| Pancreas | 1 | 0.693 | 0.922 | 1.057 | 0.951 | 0.640 | 1.069 | 1.036 | 0.940 | 1.282 | 1.253 | 1.292 | 1.106 |  |
|  |  |  |  |  |  |  |  |  |  |  |  |  |  |  |
| Melanoma | 1 | 0.361 | 0.730 | 0.789 | 0.652 | 0.537 | 0.810 | 0.559 | 1.220 | 0.622 | 0.744 | 0.970 | 0.815 |  |
|  |  |  |  |  |  |  |  |  |  |  |  |  |  |  |
| Breast | 1 | 0.766 | 1.028 | 0.898 | 0.927 | 0.895 | 0.872 | 0.962 | 1.072 | 1.117 | 1.321 | 1.122 | 1.469 |  |
|  |  |  |  |  |  |  |  |  |  |  |  |  |  |  |
| Corpus Uteri | 1 | 1.066 | 1.383 | 1.182 | 0.991 | 1.192 | 1.201 | 1.265 | 1.043 | 1.472 | 1.270 | 1.017 | 1.076 |  |
|  |  |  |  |  |  |  |  |  |  |  |  |  |  |  |
| Kidney | 1 | 0.925 | 0.855 | 0.909 | 1.200 | 1.044 | 1.021 | 0.896 | 1.120 | 0.822 | 0.963 | 0.889 | 0.782 |  |
| Ovary | 1 | 0.899 | 1.000 | 1.123 | 1.156 | 0.899 | 1.281 | 1.178 | 1.169 | 1.280 | 1.456 | 1.281 | 1.287 |  |
| Stomach | 1 | 1.304 | 0.228 | 0.662 | 0.402 | 0.520 | 0.775 | 0.860 | 0.914 | 0.699 | 0.751 | 1.330 | 0.839 |  |
| Urinary Bladder | 1 | 1.481 | 2.056 | 0.550 | 0.979 | 2.616 | 1.438 | 2.055 | 0.900 | 1.905 | 1.736 | 0.901 | 1.510 |  |
| Prostate | 1 | 1.017 | 1.205 | 1.183 | 1.445 | 1.331 | 1.102 | 1.318 | 1.065 | 1.369 | 1.255 | 1.310 | 1.117 |  |
| Liver | 1 | 0.165 | 0.168 | 0.396 | 0.289 | 0.562 | 0.144 | 0.666 | 0.690 | 0.528 | 0.632 | 0.840 | 0.573 |  |

| **SEER** |  | 2001 | 2002 | 2003 | 2004 | 2005 | 2006 | 2007 | 2008 | 2009 | 2010 | 2011 | 2012 | 2013 |
| --- | --- | --- | --- | --- | --- | --- | --- | --- | --- | --- | --- | --- | --- | --- |
| Primary cancer site |  | OR | OR | OR | OR | OR | OR | OR | OR | OR | OR | OR | OR | OR |
| All |  | 1.346 | 1.334 | 1.353 | 2.163 | 2.169 | 2.235 | 2.292 | 2.331 | 2.394 | 2.393 | 2.498 | 2.483 | 2.509 |
| Esophagus |  | 10.491 | 4.310 | 6.223 | 8.924 | 5.018 | 10.431 | 11.442 | 11.138 | 7.762 | 9.105 | 9.546 | 11.437 | 14.567 |
| Lung and Bronchus |  | 1.679 | 1.675 | 1.547 | 2.028 | 2.113 | 2.285 | 2.370 | 2.359 | 2.794 | 2.703 | 3.197 | 3.291 | 3.426 |
| Colon and Rectum |  | 1.471 | 1.520 | 1.699 | 2.063 | 1.932 | 2.151 | 2.085 | 2.274 | 2.176 | 2.410 | 2.374 | 2.339 | 2.625 |
| Pancreas |  | 1.109 | 1.350 | 0.694 | 1.249 | 1.517 | 1.829 | 1.858 | 1.942 | 2.202 | 2.448 | 1.997 | 2.434 | 2.038 |
| Melanoma |  | 0.810 | 0.767 | 0.799 | 0.965 | 0.969 | 1.060 | 1.030 | 1.008 | 0.979 | 0.937 | 1.456 | 1.438 | 1.961 |
| Breast |  | 1.251 | 1.464 | 1.206 | 1.506 | 1.527 | 1.653 | 1.662 | 1.722 | 1.769 | 1.861 | 1.935 | 1.763 | 1.916 |
| Corpus Uteri |  | 1.291 | 0.959 | 1.391 | 0.972 | 0.976 | 1.404 | 1.503 | 1.371 | 1.396 | 1.518 | 1.484 | 1.335 | 1.632 |
| Kidney |  | 1.246 | 0.965 | 1.387 | 1.377 | 1.675 | 1.155 | 1.313 | 1.459 | 1.686 | 1.407 | 1.237 | 1.687 | 1.615 |
| Ovary |  | 1.423 | 1.228 | 1.391 | 1.352 | 1.379 | 1.406 | 1.488 | 1.418 | 1.667 | 1.502 | 1.579 | 1.474 | 1.594 |
| Stomach |  | 1.081 | 1.143 | 1.410 | 1.235 | 1.180 | 1.787 | 1.666 | 2.003 | 1.905 | 1.992 | 2.415 | 2.760 | 1.585 |
| Urinary Bladder |  | 1.611 | 1.401 | 0.595 | 1.190 | 1.110 | 1.401 | 1.604 | 1.631 | 0.738 | 0.870 | 1.107 | 1.016 | 1.230 |
| Prostate |  | 1.197 | 1.197 | 0.994 | 1.258 | 1.060 | 1.083 | 1.270 | 1.145 | 1.255 | 1.137 | 1.268 | 1.388 | 1.210 |
| Liver |  | 0.537 | 0.952 | 0.925 | 0.703 | 0.588 | 0.821 | 0.935 | 0.545 | 0.336 | 0.618 | 0.970 | 0.332 | 0.346 |

**Supplemental Table 2. Changes in long term survivorship.**

Odds ratios (ORs) for survival for each cancer subtype from 1988-2013. Patients who were alive at the time of follow-up five years (60 months) after initial diagnosis were classified as survived, while those that died before the five years were classified as deceased. Patients were excluded if they had not yet survived 60 months at the time of most recent follow-up, as they had not met the 60-month requirement to be classified. OR’s were compared with the cohort of patients diagnosed in 1998 for each subtype.

**eCitations.**

1. Park HS, Lloyd S, Decker RH, Wilson LD, Yu JB. Overview of the Surveillance, Epidemiology, and End Results database: evolution, data variables, and quality assurance. *Curr Probl Cancer*. 2012;36(4):183-190. doi:10.1016/j.currproblcancer.2012.03.007

2. Casefinding Studies - SEER Quality Improvement. SEER. Accessed May 30, 2022. https://seer.cancer.gov/qi/tools/casefinding.html
