# Supplementary material for: Future trends in incidence and long-term survival of metastatic cancer in the United States
Source: Commun Med (Lond). 2023 May 27;3:76. doi: 10.1038/s43856-023-00304-x (PMC10224927; doi:10.1038/s43856-023-00304-x)
Supplement: Supplementary file 13 — Reporting Summary [file 43856_2023_304_MOESM13_ESM.pdf]

nature portfolio

Corresponding author(s): Nicholas G. Zaorsky

Last updated by author(s): Feb 21, 2023

Reporting Summary

Nature Portfolio wishes to improve the reproducibility of the work that we publish. This form provides structure for consistency and transparency in reporting. For further information on Nature Portfolio policies, see our Editorial Policies and the Editorial Policy Checklist.

Statistics

For all statistical analyses, confirm that the following items are present in the figure legend, table legend, main text, or Methods section.

n/a	Confirmed	
☐	☒	The exact sample size (*n*) for each experimental group/condition, given as a discrete number and unit of measurement
☐	☒	A statement on whether measurements were taken from distinct samples or whether the same sample was measured repeatedly
☒	☐	The statistical test(s) used AND whether they are one- or two-sided<br>*Only common tests should be described solely by name; describe more complex techniques in the Methods section.*
☒	☐	A description of all covariates tested
☒	☐	A description of any assumptions or corrections, such as tests of normality and adjustment for multiple comparisons
☐	☒	A full description of the statistical parameters including central tendency (e.g. means) or other basic estimates (e.g. regression coefficient) AND variation (e.g. standard deviation) or associated estimates of uncertainty (e.g. confidence intervals)
☐	☒	For null hypothesis testing, the test statistic (e.g. *F*, *t*, *r*) with confidence intervals, effect sizes, degrees of freedom and *P* value noted<br>*Give P values as exact values whenever suitable.*
☐	☒	For Bayesian analysis, information on the choice of priors and Markov chain Monte Carlo settings
☒	☐	For hierarchical and complex designs, identification of the appropriate level for tests and full reporting of outcomes
☒	☐	Estimates of effect sizes (e.g. Cohen's *d*, Pearson's *r*), indicating how they were calculated

Our web collection on statistics for biologists contains articles on many of the points above.

Software and code

Policy information about availability of computer code

Data collection	The data of patients with metastatic cancer, diagnosed between 1988 and 2018, was abstracted from the National Cancer Institute's Surveillance, Epidemiology, and End Results (SEER) database. For this study, the SEER 9 database was used, which represents 9% of the US population and encompasses data from 1975-2018. The SEER registry includes data on sex, age at diagnosis, race, marital status, and year of diagnosis.
Data analysis	We utilized the method developed by Yang et al. to predict future cancer incidence/mortality using age-period-cohort models. For detailed method description, please refer to the supplemental methods and sources. SEER*Stat 8.3.9, joinpoint 4.9.0.0 (available through the National Cancer Institute), and Microsoft Excel 16.16.10 (Microsoft, Redmond, WA) were used for analysis.

For manuscripts utilizing custom algorithms or software that are central to the research but not yet described in published literature, software must be made available to editors and reviewers. We strongly encourage code deposition in a community repository (e.g. GitHub). See the Nature Portfolio guidelines for submitting code & software for further information.

Data

Policy information about availability of data

All manuscripts must include a data availability statement. This statement should provide the following information, where applicable:

- Accession codes, unique identifiers, or web links for publicly available datasets
- A description of any restrictions on data availability
- For clinical datasets or third party data, please ensure that the statement adheres to our policy

Any additional information can be found within the supplementary information files or may be requested from the corresponding author. The data obtained for the current project from the SEER database is freely accessible to the public. We comply with all relevant ethical regulations. The datasets generated and analyzed during the current study are available in the SEER repository (https://seer.cancer.gov/seerstat/). The study was exempt from IRB review as these data are freely available via the National Cancer Institute SEER program. There are no participants in the study, and thus there is no consent form.

Human research participants

Policy information about studies involving human research participants and Sex and Gender in Research.

Reporting on sex and gender	Not applicable
Population characteristics	Not applicable
Recruitment	Not applicable
Ethics oversight	Not applicable

Note that full information on the approval of the study protocol must also be provided in the manuscript.

Field-specific reporting

Please select the one below that is the best fit for your research. If you are not sure, read the appropriate sections before making your selection.

☐ Life sciences ☒ Behavioural & social sciences ☐ Ecological, evolutionary & environmental sciences

For a reference copy of the document with all sections, see nature.com/documents/nr-reporting-summary-flat.pdf

Behavioural & social sciences study design

All studies must disclose on these points even when the disclosure is negative.

Study description	The study is a cross-sectional study involving only quantitative data. Patients with metastatic cancer, as coded in the SEER database, were included in the analyses.
Research sample	All patients with a metastatic cancer diagnosis, diagnosed between 1988 and 2018, for whom data was available through the SEER database were included in the study analyses. Data were abstracted from the National Cancer Institute's Surveillance, Epidemiology, and End Results (SEER) program. SEER is a network of population-based incident tumor registries from geographically distinct regions in the US, covering 9% of the US population, including incidence, survival, and limited treatment information (e.g. radiation therapy, surgery, chemotherapy). For the current analysis, the SEER 9 registries was used. The SEER registry includes data on sex, age at diagnosis, race, marital status, and year of diagnosis. The data are freely accessible to the public.
Sampling strategy	All patients coded as having metastatic cancer in the SEER database were included in the analyses (a total of 926,991 patients). This sample size was not specifically chosen, but rather included all data available in SEER at the time of this study.
Data collection	An incidence listing of patients with metastatic cancer, diagnosed between 1988 and 2018, was abstracted from the SEER program. Variables pulled included patient ID, age at diagnosis, year of diagnosis, primary cancer site. A case listing with patients ID, primary metastatic cancer site, and 5-year survival data was extracted.
Timing	Data was collect between Feb and May of 2021.
Data exclusions	Patients diagnosed with cancer only through autopsy or death certificate were excluded. For survival data, patients with follow-up time less than 60 months were excluded from the analysis because it was not possible to determine whether or not they would eventually be classified as long-term survivors. Thus, the patient population included (i) deceased patients with survival times less than 60 months and (ii) all patients with follow-up times greater than or equal to 60 months.
Non-participation	Because the study design is using retrospective data, non-participation is not applicable.

Randomization	Because the study design is using retrospective data, randomization is not applicable.

Reporting for specific materials, systems and methods

We require information from authors about some types of materials, experimental systems and methods used in many studies. Here, indicate whether each material, system or method listed is relevant to your study. If you are not sure if a list item applies to your research, read the appropriate section before selecting a response.

Materials & experimental systems

n/a	Involved in the study
☒	☐ Antibodies
☒	☐ Eukaryotic cell lines
☒	☐ Palaeontology and archaeology
☒	☐ Animals and other organisms
☒	☐ Clinical data
☒	☐ Dual use research of concern

Methods

n/a	Involved in the study
☒	☐ ChIP-seq
☒	☐ Flow cytometry
☒	☐ MRI-based neuroimaging
